# Postlaryngectomy supraglottic stenosis revealed by three-dimensional computed tomography reconstruction: A case report

**DOI:** 10.1097/MD.0000000000028769

**Published:** 2022-02-04

**Authors:** JinYoung Chon, SungJin Hong, SangHoon Lee, MinJung Shin, SeungHee Cha, JiYung Lee

**Affiliations:** Department of Anesthesiology and Pain Medicine, College of Medicine, The Catholic University of Korea, Seoul, Korea.

**Keywords:** asthma, computed tomography, supraglottic stenosis, three–dimensional reconstruction

## Abstract

**Rationale:**

Supraglottic stenosis is a rare cause of airway obstruction. It can be induced by radiation, trauma, autoimmune diseases, or caustic exposure, and is often misdiagnosed as asthma. Detailed airway information is necessary to re-establish the normal functioning of the airway.

**Patient concerns:**

A 78-year-old woman with severe dyspnea and hypercarbia was scheduled for surgery to resolve airway obstruction, previously known as supraglottic stenosis.

**Diagnoses:**

To determine the exact internal shape of the stenotic lesion, we reconstructed three dimensional computed tomography (CT) images depicted a tubular supraglottic stenosis.

**Interventions:**

The patient underwent tracheotomy under monitored anesthesia care and local anesthesia, followed by general anesthesia. For long-term management of the patient, the otorhinolaryngologist excised the supraglottic stricture via micro-laryngeal surgery using a CO_2_ laser and applied mitomycin to prevent further obstruction.

**Outcomes:**

The patient recovered uneventfully after anesthesia, and symptom due to supraglottic stenosis was improved.

**Lessons:**

During airway management of patients with postlaryngectomy supraglottic stenosis, three-dimensional reconstructed computed tomography images facilitate airway configuration in addition to endoscopy and other radiological findings.

## 1 Introduction

Supraglottic stenosis is a rare condition caused by acute or chronic swelling of the supraglottic larynx. It can be induced by inflammation or irradiation, chronic scarring due to prolonged instrumentation, autoimmune disease, gastro-esophageal reflux disease, or idiopathic causes.^[1–3]^ It results in asthma symptoms, warranting a differential diagnosis.^[4,5]^

In patients with airway obstruction such as supraglottic stenosis, accurate assessment of the airway is essential for airway reestablishment. Although it may not facilitate patient management, three-dimensional reconstruction of head and neck CT images provides precise structural information on the larynx and trachea. Initially used in the fields of otolaryngology and plastic or dental surgery, 3D-CT may be a useful modality for airway management.^[6,7]^ Herein we describe the diagnosis and treatment of a patient with postlaryngectomy supraglottic stenosis, and present 3D reconstructed images of supraglottic stenosis for clinical application in anesthesiology.

## 2 Case presentation

A 78-year-old woman was brought to the operating room for tracheotomy under local anesthesia due to frequent asthma attacks and progressive hypercarbia for the past 3 days. She was 152.1 cm tall and weighed 53.60 kg, corresponding to a body mass index of 23.17.

Nineteen years ago, she had undergone supraglottic laryngectomy and lymph node dissection on the right side of her neck for squamous cell carcinoma involving right false vocal fold of the larynx (T2N0M0), without chemotherapy or radiotherapy. She had developed arytenoid swelling and supraglottic obstruction 4 years previously, which were detected via laryngoscopy. At that time, she had lung cancer and underwent open thoracotomy and right lower lobectomy with lymph node dissection. Anesthetic records revealed that she had undergone tracheotomy under local anesthesia, followed by general anesthesia and lung isolation with Arndt endobronchial blocker. Three years ago, she had been diagnosed with pulmonary tuberculosis and treated with anti-tuberculosis medication for 6 months.

**Figure 1:**
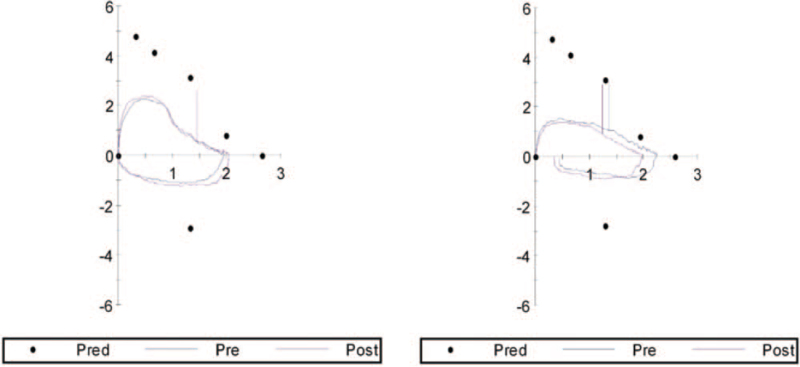
Flow-volume loops in pulmonary function testing showing progressive extrathoracic airway obstruction. There were no significant responses following administration of bronchodilators. Left: Loop acquired in the previous year. Right: Loop acquired in the current year.

Over the last 2 years, she had frequently been admitted to several hospitals due to dyspnea, and treated for asthma. Her current medications included doxofylline, erdosteine, candesartan, megestrol acetate suspension, fluticasone/formoterol inhaler, and tiotropium. The most recent pulmonary function test depicted flow volume loops suggestive of fixed extrathoracic obstruction that was more advanced than it had been the previous year (Fig. 1).

Her arterial blood gas analysis (ABGA) results are as follows: pH 7.32, PaCO_2_ 63.4 mm Hg, PaO_2_ 92.3 mm Hg, HCO_3-_ 32.2 mmol/L, oxygen saturation 96.6%. Other laboratory values were unremarkable.

**Figure 2:**
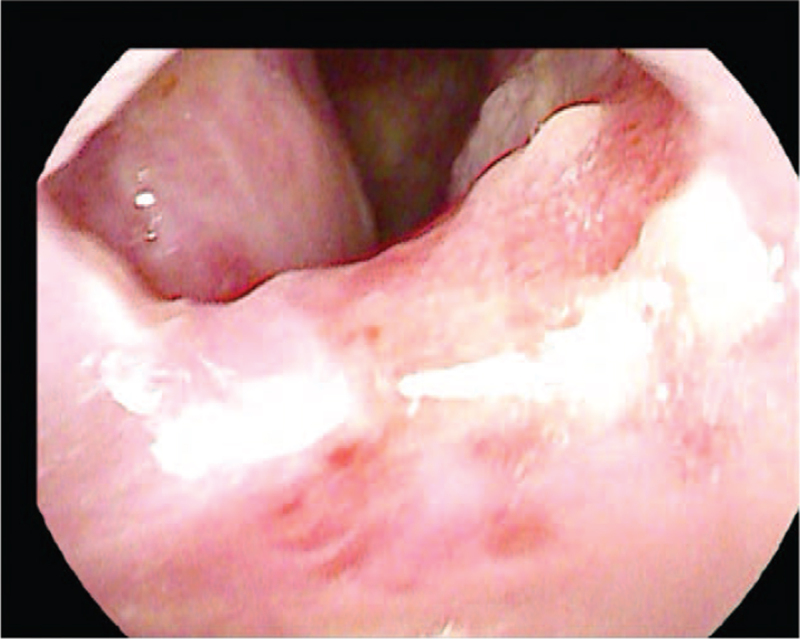
Preoperative endoscopic view showing obstructive lesion hiding the laryngeal inlet.

**Figure 3:**
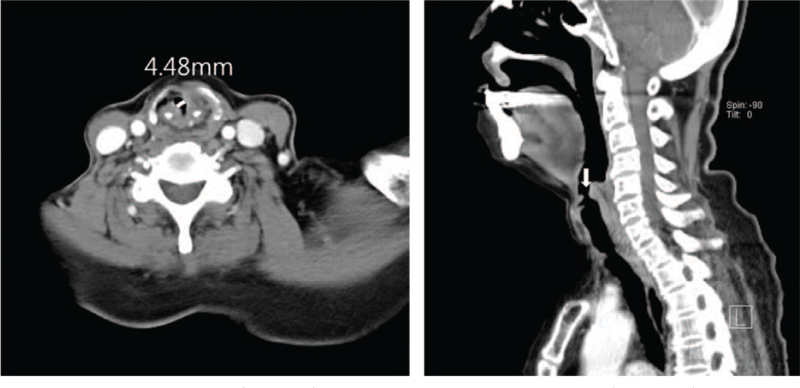
Preoperative axial computed tomography view (left, arrow), and sagittal computed tomography view (right, arrow) showing a very small opening into the larynx (supraglottic obstruction).

**Figure 4:**
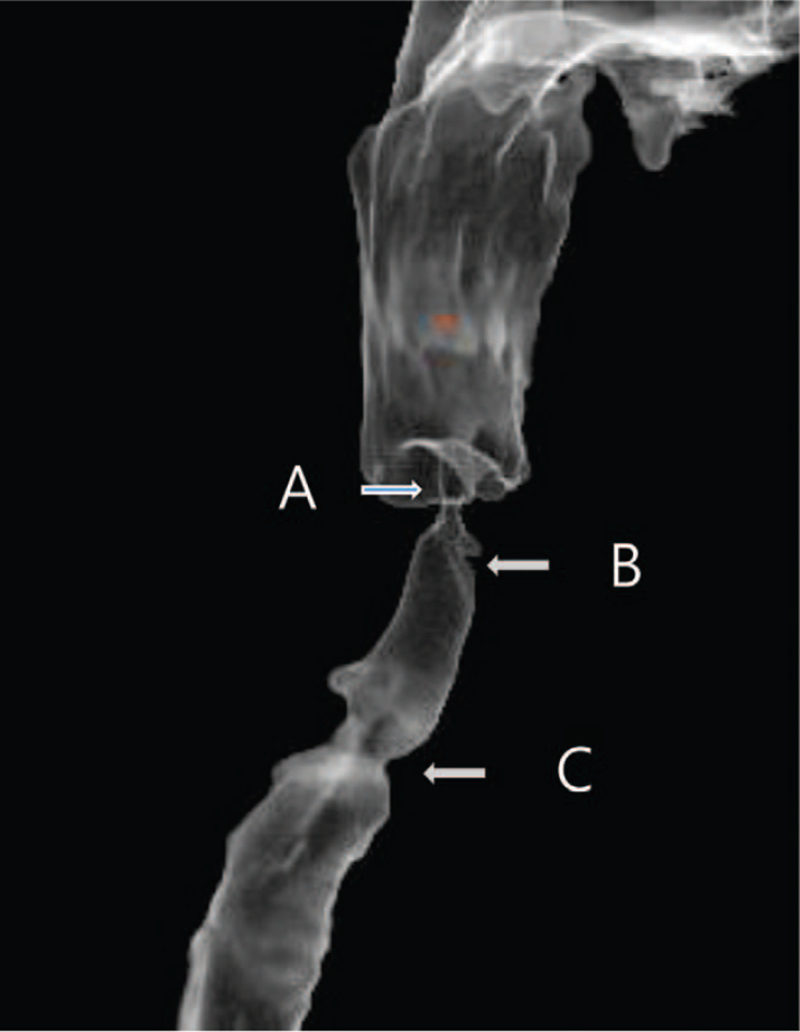
Three-dimensional reconstructed computed tomography image showing supraglottic stenosis in addition to tracheal narrowing during a previous tracheotomy. (A). supraglottic stenosis. (B). Glottis. (C). Prior tracheotomy site.

In the previous consultation for general anesthesia referral, the severe obstruction above the glottis was shown in endoscopic view and CT scans (Figs. 2 and 3). Those images depicted only a small elliptical opening with a short axis measuring <5 mm in diameter between the pharynx and vocal cord. We requested 3D CT reconstruction and informed that intubation was not possible as a result. These investigations suggested that it was not a membranous opening, but a tubular narrowing proximal to the glottis (Fig. 4).

The patient was transferred with oxygen supplementation at a rate of 3 L/min via a nasal cannula, but became severely dyspneic and diaphoretic, and was unable to talk or lie down after entering the operating room. Her vital signs were as follows: blood pressure 191/130 mm Hg, heart rate 130 beats/min with frequent ventricular premature complex on electrocardiography, and oxygen saturation (SpO_2_) 90% to 98%. The otorhinolaryngologist called the anesthesia team urgently for patient management. The patient was instructed to inhale Ventolin (albuterol sulfate), but it was ineffective. The results of ABGA were as follows: pH 7.28, PCO_2_ 69.4 mm Hg, PO_2_ 179.3 mm Hg, HCO_3_^-^ 31.7 mmol/L, and a base excess of 2.7 mmol/L. Because of aggravation of hypertension and tachycardia of up to 201/90 mmHg and 129 beats/min, respectively, esmolol 10 mg was injected. Intravenous aminophylline hydrate 250 mg was injected slowly followed by continuous infusion at a rate of 0.4 mg/kg/h. Hydrocortisone 100 mg and methylprednisolone 125 mg were also injected. Within 5 minutes after aminophylline administration, her dyspnea improved dramatically, and she was able to lie down on the table. Her vital signs were blood pressure 137/85 mmHg, heart rate 111 beats/min, and SpO_2_ 98%. Tracheotomy was rapidly performed under local anesthesia. For analgesia, fentanyl 50 μg was injected. Tracheotomy for the third tracheal ring was conducted with a tracheostomy tube with an inner diameter of 6.0 mm with a cuff. During the tracheal intubation, propofol 60 mg and rocuronium bromide 40 mg were injected, and the tracheotomy tube was connected to the anesthetic circuit for general anesthesia with air-oxygen-sevoflurane. After induction of general anesthesia, ABGA revealed the following: pH 7.411, PCO_2_ 44.4 mm Hg, PO_2_ 430.0 mm Hg, HCO_3_^-^ 27.6 mmol/L, and base excess 2.5 mmol/L.

**Figure 5:**
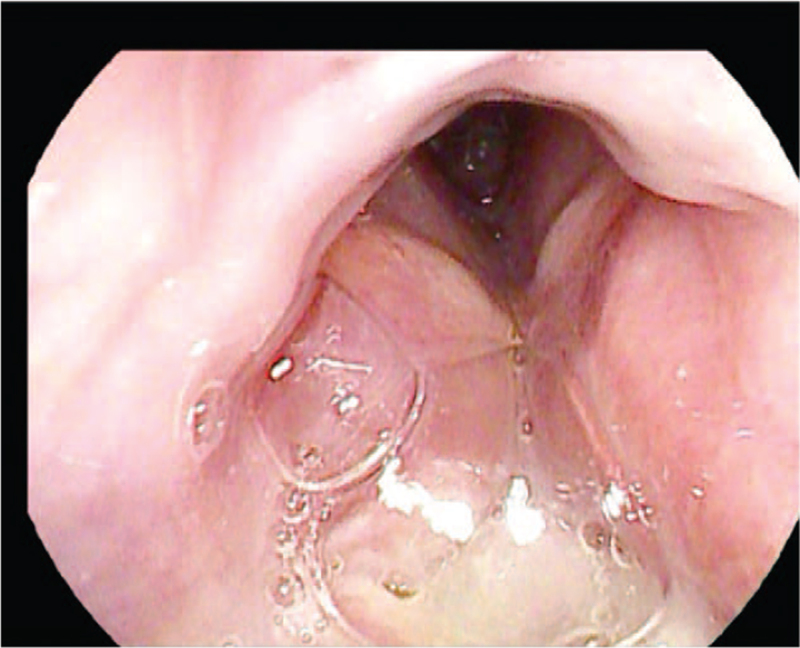
Postoperative endoscopic image showing marked widening of the laryngeal inlet after micro-laryngeal surgery with a CO_2_ laser.

The otorhinolaryngologist excised the supraglottic stricture via micro-laryngeal surgery using a CO_2_ laser and applied mitomycin to prevent further obstruction. After the completion of surgery, neuromuscular function was recovered by sugammadex 200 mg. The patient was transferred to postanesthesia care unit after recovery of spontaneous respiration and disconnection from anesthetic circuit. She was discharged 17 days later and free from dyspnea due to airway obstruction. Postoperative endoscopic evaluation depicted significant widening of the stenosis (Fig. 5).

This case report was approved by the Institutional Review Board (IRB) of St. Mary's Hospital, The Catholic University of Korea (approval number SC19ZESE0113). The patient provided written informed consent for publication of this report.

## 3 Discussion

In patients with airway obstruction such as supraglottic stenosis, accurate assessment of the airway is essential for airway reestablishment. Endoscopic and radiologic evaluation is generally used for the diagnosis of airway obstruction. In the present case however, the nature of supraglottic stenosis from the opening of the laryngopharynx to the vocal cord could not be explained. The opening could have been a hole in the membrane or the end of a tubular segment. The feature of the stenotic lesion is an important issue for anesthesiologists with regard to predicting the progress of endotracheal intubation in patients who require orotracheal intubation. In the present case, the supraglottic obstruction appeared to be a small elliptical opening with a minor axis of less than 5 mm based on endoscopic findings and CT, which suggested potential space between the membrane and the glottis. Intubation through the opening does not guarantee accurate targeting to the glottis in such cases because the field of vision is limited. Notably however, the reconstructed 3D images of the laryngotracheal tree from head and neck CT showed that the supraglottic obstruction was a tubular segment, resulting in stenosis. As a result, we were able to convince the surgical team that orotracheal intubation to conduct general anesthesia was impossible. Airway evaluation using 3D reconstructed images should facilitate planning of an airway operation and anesthesia.^[7]^

Airway obstruction can be misdiagnosed as asthma because both conditions result in similar symptoms such as dyspnea and wheezing.^[4]^ In the current patient, supraglottic obstruction was first noticed 14 years after supraglottic laryngectomy because of loss to long–term follow-up. Because of the similarity of symptoms, it was not clear whether this patient's dyspnea was due to supraglottic obstruction, asthma, or both. Sudden exacerbation of severe dyspnea after admission to the operating room suggested a possible asthma attack. Notably however, a lack of response to albuterol inhalation that was conducted for the treatment and differential diagnosis of asthma resulted in confusion. Even though she had been treated with albuterol on numerous prior occasions, it was ineffective at this time. Based on the fact that intravenous aminophylline was effective, it was suggested that the patient may not have inhaled adequate amounts of albuterol for delivery to the tracheobronchial tree because of severe dyspnea and supraglottic obstruction.

Tam et al^[8]^ reported the use of high-flow nasal cannula (HFNC) during the widening of stenosis via laser without intubation in a patient with pharyngeal-supraglottic stenosis and scarring from the epiglottis to the pharyngeal walls, and a cicatricial scar band. In total apnea, the duration of oxygenation with HFNC lasted 26 minutes. The surgery focused on transection of the scar tissue before intubation while oxygen was supplied via HFNC, irrespective of the ABGA data. Although Ebeling and Riccio^[9]^ reported that it was not certain whether HFNC was sufficient for ventilation under apneic conditions, Kim et al^[10]^ reported that it improved oxygenation and ventilation in patients with acute respiratory failure and hypercapnia under spontaneous ventilation. Unfortunately, the current patient already had severe hypercarbia and urgently required ventilation. The application of HFNC was ruled out. However, HFNC is a promising and useful tool in patients with airway obstruction including supraglottic stenosis.

Bronchoscopic intubation is another potential option for airway establishment, but it was not desirable in the present case. The stenosis was narrow enough to pass only tubes less than 5 mm in outer diameter and as the bronchoscope should pass through that tube, there is little space for ventilation. However, if a patient with supraglottic stenosis is stable, well-oxygenated, and ventilated, bronchoscopic intubation using a pediatric endoscope is appropriate.

In this case report, we have described the case of a patient with post-laryngectomy supraglottic stenosis with a superimposed asthma attack who was treated effectively. During airway management of a patient with post-laryngectomy supraglottic stenosis, 3D reconstructed CT images facilitate airway configuration in addition to endoscopy and other radiological findings.

## Author contributions

**Conceptualization:** JinYoung Chon.

**Data curation:** JinYoung Chon.

**Investigation:** MinJung Shin, SeungHee Cha.

**Supervision:** SungJin Hong.

**Visualization:** SangHoon Lee.

**Writing – original draft:** JiYung Lee.

**Writing – review & editing:** JiYung Lee.
